# Preeclampsia is Associated with Sex-Specific Transcriptional and Proteomic Changes in Fetal Erythroid Cells

**DOI:** 10.3390/ijms20082038

**Published:** 2019-04-25

**Authors:** Zahra Masoumi, Gregory E. Maes, Koen Herten, Álvaro Cortés-Calabuig, Abdul Ghani Alattar, Eva Hanson, Lena Erlandsson, Eva Mezey, Mattias Magnusson, Joris R Vermeesch, Mary Familari, Stefan R Hansson

**Affiliations:** 1 Division of Obstetrics and Gynecology, Department of Clinical Sciences Lund, Lund University, Klinikgatan 28, 22184 Lund, Sweden; eva.hansson@med.lu.se (E.H.); lena.erlandsson@med.lu.se (L.E.); 2 Laboratory for Cytogenetics and Genome Research, Center for Human Genetics, KU Leuven, Herestraat 49, 3000 Leuven, Belgium; gregory.maes@kuleuven.be (G.E.M.); koen.herten@kuleuven.be (K.H.); joris.vermeesch@uzleuven.be (J.R.V.); 3 Department of Human Genetics, Centre for Human Genetics, University Hospital Leuven, KU Leuven, Herestraat 49, 3000 Leuven, Belgium; 4 Genomics Core, UZ Leuven, Herestraat 49, 3000 Leuven, Belgium; alvaro.cortes@uzleuven.be; 5 Department of Hematology and Transfusion Medicine, Lund University, Klinikgatan 28, 22184 Lund, Sweden; abdul_ghani.alattar@med.lu.se; 6 Department of Molecular Medicine and Gene Therapy, Lund University, Sölvegatan 17, 22184 Lund, Sweden; mattias.magnusson@med.lu.se; 7 Adult Stem Cell Section, National Institute of Dental and Craniofacial Research, National Institutes of Health, 9000 Rockville Pike, Bethesda, MD 20892, USA; MezeyE@nidcr.nih.gov; 8 School of Biosciences, Bldg 4, University of Melbourne, Parkville 3010, Australia; m.familari@unimelb.edu.au; 9 Lund University, Skåne University Hospital, Obstetrics and Gynecology, Department of Clinical Sciences Lund, Klinikgatan 28, 22184 Lund, Sweden; stefan.hansson@med.lu.se

**Keywords:** preeclampsia, hematopoietic stem/progenitor cells, umbilical cord blood, erythropoiesis

## Abstract

Preeclampsia (PE) has been associated with placental dysfunction, resulting in fetal hypoxia, accelerated erythropoiesis, and increased erythroblast count in the umbilical cord blood (UCB). Although the detailed effects remain unknown, placental dysfunction can also cause inflammation, nutritional, and oxidative stress in the fetus that can affect erythropoiesis. Here, we compared the expression of surface adhesion molecules and the erythroid differentiation capacity of UCB hematopoietic stem/progenitor cells (HSPCs), UCB erythroid profiles along with the transcriptome and proteome of these cells between male and female fetuses from PE and normotensive pregnancies. While no significant differences were observed in UCB HSPC migration/homing and in vitro erythroid colony differentiation, the UCB HSPC transcriptome and the proteomic profile of the in vitro differentiated erythroid cells differed between PE vs. normotensive samples. Accordingly, despite the absence of significant differences in the UCB erythroid populations in male or female fetuses from PE or normotensive pregnancies, transcriptional changes were observed during erythropoiesis, particularly affecting male fetuses. Pathway analysis suggested deregulation in the mammalian target of rapamycin complex 1/AMP-activated protein kinase (mTORC1/AMPK) signaling pathways controlling cell cycle, differentiation, and protein synthesis. These results associate PE with transcriptional and proteomic changes in fetal HSPCs and erythroid cells that may underlie the higher erythroblast count in the UCB in PE.

## 1. Introduction

Preeclampsia (PE) is a pregnancy related disorder that remains a major cause of maternal and fetal mortality by affecting 3% to 8% of pregnancies worldwide [1]. Although it is diagnosed after 20 weeks of gestation, based on the maternal symptoms, such as high blood pressure and proteinuria [2], the events leading to PE may be triggered during trophoblast invasion and embryonic implantation [3]. Insufficient trophoblast invasion into the decidua and the spiral arteries leads to dysfunctional placentation, altered placental blood flow, subsequent hypoxia, and cell senescence [4,5,6,7,8]. Changes in cellular metabolism, increased placental oxidative stress, and placental barrier damage lead to leakage of placental and fetal factors into the maternal circulation [5,9]. One of these factors is free fetal hemoglobin (HbF), which is found to be elevated in the maternal circulation as early as the first trimester in women who later develop PE [10]. Free hemoglobin triggers inflammation, vasoconstriction, and tissue damage [11], and particularly impairs the placenta barrier [12].

The negative consequences of a PE pregnancy affect the mother as well as the developing fetus [13,14,15,16]. Our group has previously demonstrated higher levels of total hemoglobin in the arterial and venous UCB [17], suggesting possible accelerated fetal erythropoiesis. Higher erythroblast count in the UCB is a common observation in PE pregnancies [18,19,20,21] that has been linked to elevated erythropoiesis induced by hypoxia and enhanced fetal erythropoietin (EPO) levels [22,23,24,25]. However, previous studies investigating in vitro colony formation of UCB HSPCs have not demonstrated any significant differences in the erythroid differentiation capacity of the cells obtained from PE vs. normotensive pregnancies [26,27]. This suggests that a mechanism other than hypoxia-induced EPO-dependent enhanced fetal erythropoiesis may affect erythroid maturation, underlying the higher erythroblast count documented in the UCB of PE pregnancies. For instance, levels of glucose, glucose/insulin ratio [28,29], lactic acid [30], and inflammatory cytokines, such as tumor necrosis factorα (TNFα) [31], are reported to be altered in the fetuses from PE pregnancies. All these factors are significant in regulating erythropoiesis and erythroid maturation [32,33,34,35,36]. However, the effects of these changes on the molecular pathways that control HSPC differentiation and late erythroid maturation in the fetus have not been investigated in detail.

Another important factor in regulating fetal hemoglobin levels is fetal sex [37,38], which also determines the onset of the disease and severity of maternal symptoms in PE [39,40] as well as maternal adaptation to pregnancy [41]. Sex-specific fetal responses in PE have been assessed in both an animal model [42] and in humans [43,44,45,46]. However, these studies are limited to comparing clinical parameters, such as blood pressure, height, and weight, indicating the importance of further analysis to investigate the molecular changes.

The focus of this study was to evaluate any changes in the migration/homing or differentiation capacity of fetal HSPCs by analyzing the expression of surface adhesion molecules (SAMs), the transcriptome, and the erythroid differentiation capacity of UCB hematopoietic stem/progenitor cells (HSPCs) in fetuses from PE pregnancies. Moreover, the proteomic profile of in vitro differentiated erythroid cells was studied to explore possible differences in erythroid maturation. Finally, using an established flow cytometry analysis [47], the profile of terminally differentiating erythroblasts in the UCB was compared between PE and normotensive pregnancies to explore any alterations in fetal erythroid populations. In addition, transcriptome analysis on isolated erythroid cells was performed, with particular attention to sex-specific differences, using SE50bp RNA sequencing.

## 2. Results

### 2.1. Preeclampsia Does Not Alter Migration/Homing or Differentiation Capacity of UCB HSPCs

**Figure 1:**
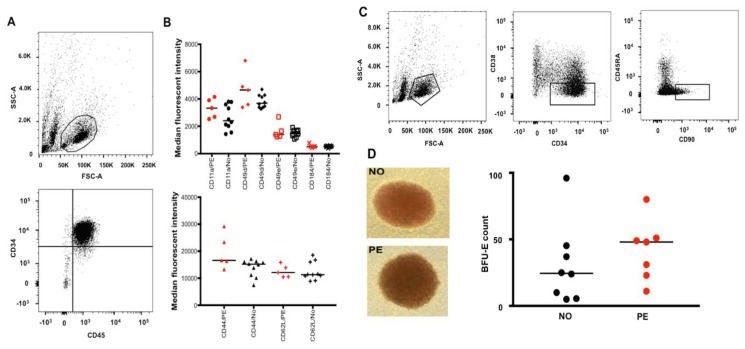
Flow cytometry analysis and isolation of UCB HSPCs and HSCs as well as assessment of surface adhesion molecules (SAMs) and in vitro erythroid differentiation capacity of the cells isolated from PE vs. normotensive (NO) pregnancies. (**A**) Flow cytometry analysis showing the UCB HSPC population gated based on size and granularity (FSC-A and SSC-A) and CD34^+^ CD45^+^ expression. (**B**) Demonstrating the median fluorescent intensity (MFI) for various SAMs in red (PE, *n* = 5) and black (NO, *n* = 10); despite large differences in some MFI values, the differences were not statistically significant. (**C**) Flow cytometry analysis of the HSC population from UCB samples; the population was gated (from left to right) based on size and granularity followed by CD34^+^, CD38^lo^, and CD45RA^−^, CD90^+^ expression. As previously reported by others, the CD34^+^ CD38^lo^ population was very small in the majority of our samples. This specific individual sample with a large CD34^+^ CD38^lo^ population was particularly chosen for specifically visualizing a clearly distinct CD34^+^ CD38^lo^ CD45RA^−^ and CD90^+^ population in the figure. (**D**) Example of BFU-Es in culture (10× magnification) from normotensive (*n* = 8) and PE (*n* = 7) samples after the UCB CD34^+^ cells were cultured for 14 days. No significant difference was observed BFU-E count comparison between PE and normotensive groups.

To study the effect of PE on migration/homing of fetal HSPCs, the expression of known surface adhesion molecules (SAMs) was analyzed on the UCB CD34^+^ CD45^+^ cells (Figure 1A). The expression of CD49d, CD49e, CD184 (CXCR4), and CD11a (the upper panel Figure 1B) as well as CD44 and CD62L (L-selectin) (the lower panel Figure 1B) were not significantly different between the PE and normotensive groups (flow cytograms demonstrated in Supplementary Figure S1). In addition, the frequency of viable hematopoietic stem cell-enriched cells (HSCs) expressing specific markers (Table 1) was determined using flow cytometry (Figure 1C). The number of CD34^+^ cells or the HSCs per mL of UCB obtained from each sample was not significantly different between the PE and normotensive samples.

To investigate whether PE altered the erythroid differentiation capacity of fetal HSPCs, resulting in enhanced erythropoiesis and UCB erythroblast count, CD34^+^ cells from normotensive and PE UCB samples were isolated and used in a colony formation assay. Despite a large difference in the median values between the groups, the number of burst forming units-erythroid (BFU-Es) showed no significant difference (Figure 1D).

### 2.2. Preeclampsia Affects the Gene Expression in UCB HSPCs

**Figure 2:**
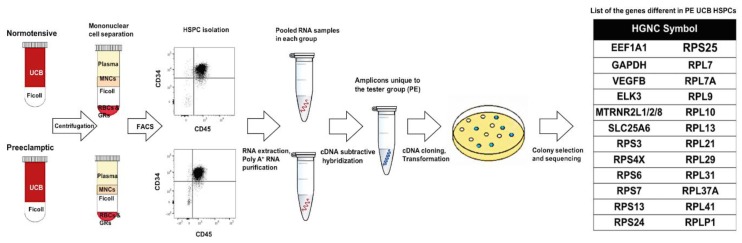
Gene expression analysis in the UCB HSPCs using cDNA subtractive hybridization. The procedure is demonstrated from separating mononuclear cells (MNCs) from the umbilical cord blood (UCB) and isolating hematopoietic stem/progenitor cells (HSPCs) to the final list of genes that were found to be different in PE. The HSPCs (CD34^+^ CD45^+^) were sorted during SAM analysis and were used in this experiment (PE, *n* = 5 and NO, *n* = 10).

Since there was no difference in the migration/homing or erythroid differentiation capacity of the UCB HSPCs (CD34^+^ CD45^+^ cells) that could explain the higher UCB erythroblast count documented in PE [18,19,20,21], cDNA subtractive hybridization was carried out to elucidate possible gene expression differences that might affect the maturation of erythroblasts. To perform cDNA subtractive hybridization, CD34^+^ CD45^+^ cells from normotensive and PE UCB samples were used as driver and tester groups, respectively. Sequencing of the differential fragments resulted in 26 protein-coding genes (Figure 2). Predictions by String suggested that the eukaryotic translation elongation factor 1 alpha 1 (EEF1A1) interacted with glyceraldehyde 3-phosphate dehydrogenase (GAPDH) as well as several ribosomal proteins (RPs). The pathways of significance based on GSEA are presented in Supplementary Table S1. The RPs were also associated with significant hematological phenotypes, such as increased mean corpuscular volume, macrocytic anemia, persistence of HbF, and reticulocytopenia, as determined by ToppFun (false discovery rate (FDR) < 0.01).

### 2.3. Preeclampsia Is Associated with Changes in Metabolic and Protein Synthesis Pathways of In Vitro Differentiated Erythroid Cells

**Figure 3:**
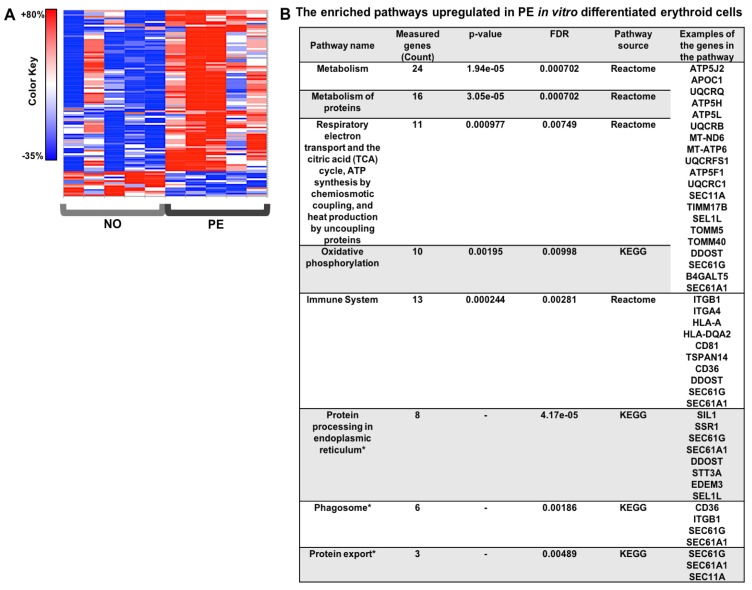
The proteomics analysis heat map and the enriched pathways in the in vitro differentiated erythroid colonies. (**A**) The heat map for the significantly differentially expressed proteins in PE (*n* = 5) vs. normotensive (*n* = 5) in vitro differentiated erythroid cells. (**B**) The enrichment analysis in the gene set analysis in CPDB human network was performed based on the protein average fold ratio in PE and normotensive samples.

To investigate whether changes in ribosomal and metabolic pathways in the HSPCs affected late erythroid maturation steps in fetuses from PE pregnancies, proteomics analysis was performed using TMT-mass spectrometry on in vitro differentiated erythroid cells. After mapping the peptide sequences to proteins, 6222 proteins were detected (FDR ≤0.01) (Supplementary Table S2). At a threshold of fold change ≥20% and *p* value ≤0.05, a total of 90 proteins were increased and 14 proteins were decreased in PE vs. normotensive in vitro differentiated erythroid cells (Supplementary Table S2). The heat map of the differentially expressed proteins and the enriched pathways predicted by GSEA are presented in Figure 3. The protein–protein interaction network and the connection between the enriched pathways are presented in Supplementary Figure S2. The affected pathways were mainly related to ATP production (oxidative phosphorylation and the TCA cycle), as well as protein synthesis, transport, and metabolism (Figure 3).

### 2.4. Preeclampsia Does Not Alter the UCB Profile of Terminally Differentiating Erythroblasts 

**Figure 4:**
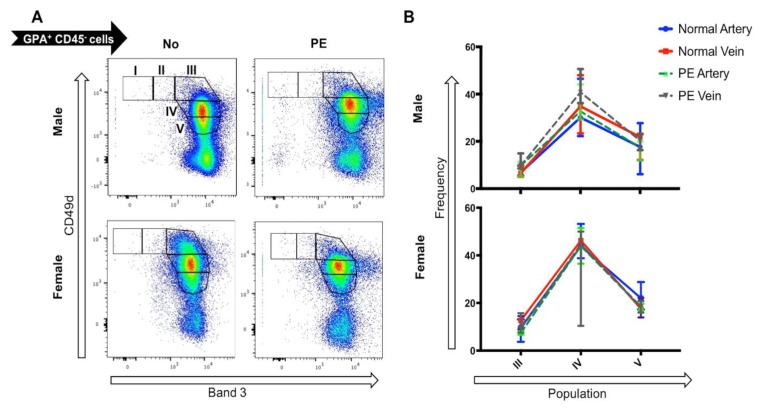
Comparison of the erythroid progenitor profile in the umbilical cord blood (UCB) from PE (*n* = 6) and normotensive (*n* = 7) pregnancies. (**A**) The flow cytometry profile of CD45^−^ GPA^+^ cells expressing CD49d (Integrin a4) and Band 3 protein during their maturation from proerythrocytes (CD49d^+^ Band 3^−^) to mature erythrocytes (Cd49d^−^ Band 3^+^). (**B**) No significant differences were observed between arterial or venous UCB from male or female in PE vs. normotensive pregnancies.

Considering that the in vitro analyses indicated no changes in molecular pathways rather than erythroid cell production, the frequency of various stages of terminally differentiating erythroid cells was investigated in the UCB erythroblasts between male and female fetuses from PE and normotensive pregnancies. The viable single cells were gated based on GPA and CD45 expression. The CD45^−^, GPA^+^ erythroid population was analyzed for surface expression of CD49d and Band 3 to evaluate the terminal erythroid differentiation stages of the UCB erythroblasts (Figure 4A). The erythroid precursors present in the samples were predominately basophilic erythroblasts II to orthochromatic erythroblasts. Comparing the erythroid profile of the samples, no significant differences were observed between the venous or arterial UCB from PE or normotensive pregnancies in male nor female fetuses (Figure 4B). 

### 2.5. Gene Expression Differences between Male vs. Female Samples Are Irrespective of Pregnancy Outcome 

**Figure 5:**
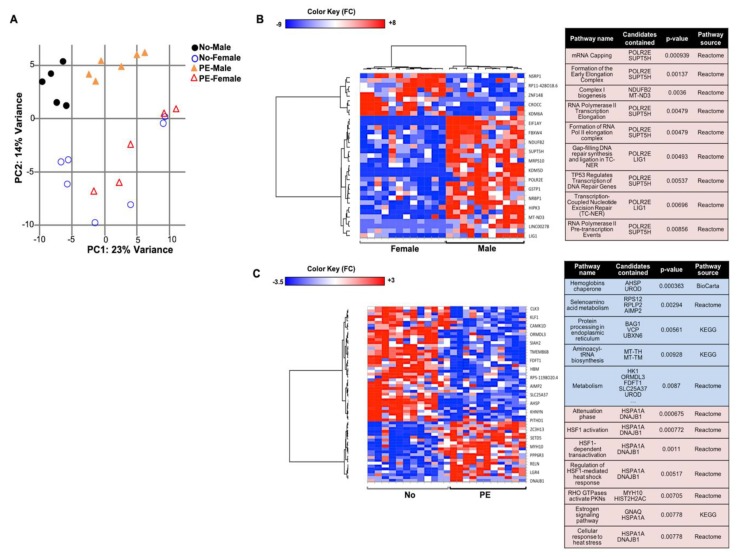
Primary gene expression analysis of the UCB erythroid progenitors. (**A**) A distinct clustering was observed between male and female samples in the principal component analysis (PCA) plot. Also, among the PE (*n* = 6) and normotensive (*n* = 7) samples, a clearer clustering was observed in the male compared to female fetuses. The heat maps of the differentially expressed genes and the related pathways are presented when comparing (**B**) male vs. female and (**C**) PE vs. normotensive samples. Shades of blue and red indicates down- or up-regulated genes/pathways, respectively.

The absence of a significant increase in the frequency of immature erythroid cells in PE UCB samples was in line with the results from the in vitro differentiation cultures. Therefore, further RNA-sequencing analysis was performed to explore possible changes in the molecular pathways that may explain the higher erythroblast count. Gene expression of arterial vs. venous erythroid cells did not differ significantly (Supplementary Figure S3). Sample clustering by principal component analysis (PCA) indicated a major difference in gene expression in the male vs. female erythroid cells irrespective of pregnancy outcome (Figure 5A). A total of 35 genes were determined by EdgeR and Deseq2 to be differentially expressed (DE) in male vs. female fetuses, affecting pathways, such as RNA transcription (Supplementary Table S3) (Figure 5B). A more distinct clustering of PE vs. normotensive samples was observed in the samples from male fetuses (Figure 5A). Based on the DE genes confirmed by Deseq2 and EdgeR, 40 genes that affected metabolism and protein processing in endoplasmic reticulum (ER)/vesicle trafficking were downregulated in PE (Figure 5C). In addition, 21 genes that were involved in pathways, such as heat shock response and protein kinase activation by RHO GTPases, were upregulated (Figure 5C).

### 2.6. Effects of PE on Gene Expression in UCB Erythroid Cells Are Sex-Specific

**Figure 6:**
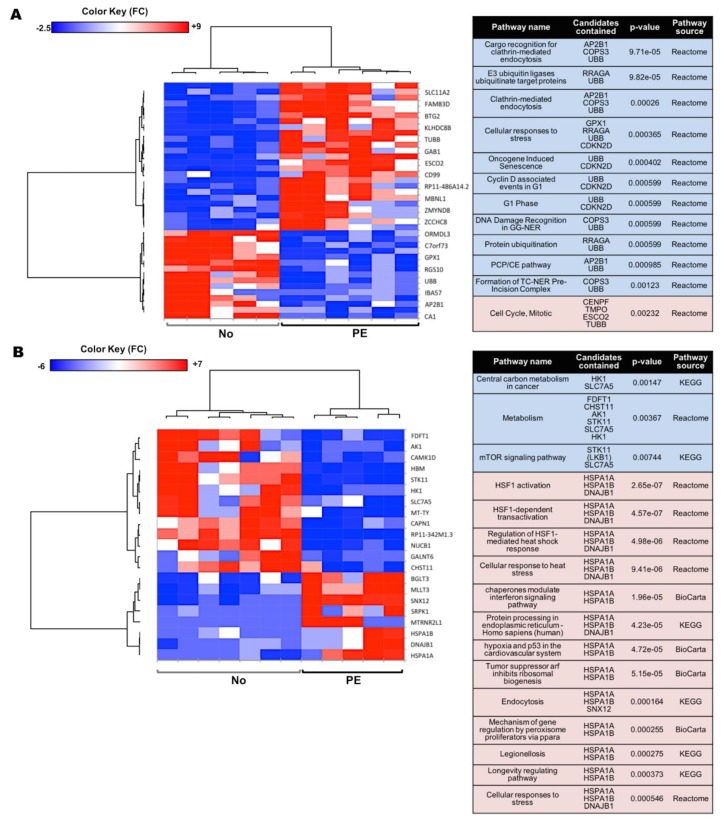
Sex-specific transcriptome comparison in the UCB erythroid progenitors from PE vs. normotensive pregnancies. (**A**,**B**) The heat maps of the differentially expressed genes and the related pathways are presented when comparing PE vs. normotensive sample from (**A**) male and (**B**) female fetuses. Shades of blue and red indicate down- or up-regulated genes/pathways, respectively.

Taking into account the sex-specific clustering of the samples from the RNA-sequencing analysis, the effect of PE on male and female samples was analyzed separately. In the males, a total of 40 DE genes were determined in PE vs. normotensive groups by Deseq2 and EdgeR (Supplementary Table S3). The affected pathways included endocytosis, protein ubiquitination, regulation of cell cycle, and convergent extension, i.e., the process of cell lengthening and narrowing along one axis (Figure 6A). Among the females, a total of 21 DE genes were confirmed in the PE group using Deseq2 and EdgeR (Supplementary Table S3). The altered pathways included metabolism, mTOR signaling, and cellular response to stress (via heat shock proteins) (Figure 6B).

### 2.7. Data Archiving

All data generated or analyzed during this study are included in this published article (and its Supplementary Information files). All the datasets generated during the current study are available on European Nucleotide Archive (https://www.ebi.ac.uk/ena) under accession number PRJEB27744 and PRIDE archive (https://www.ebi.ac.uk/pride/archive) under accession number PXD010364.

## 3. Discussion

This study focused on analyzing the changes in the molecular pathways regulating HSPCs and erythroid differentiation in fetuses from PE pregnancies. Our results were in agreement with previous reports [27,48] demonstrating no significant differences in the frequency of UCB HSPCs or the expression of the SAMs on these cells, despite originating from PE or normotensive pregnancies. In addition, the absence of significant differences in the number of BFU-Es produced during in vitro differentiation of the UCB CD34^+^ cells from PE and normotensive groups in our samples was in accordance with previous reports [26,27]. Interestingly, both function and expression of SAMs on HSPCs are precisely regulated during embryonic development [49,50,51] as well as lineage differentiation [47,52,53]. Thus, the absence of significant differences in SAM expression and colony formation imply that PE may not cause significant changes in fetal HSPC migration/homing or intrinsic differentiation capacity.

Investigation of transcriptional changes demonstrated differences in gene expression in UCB HSPCs in PE vs. normotensive pregnancies. Many of the detected mRNAs were those of ribosomal proteins (RPs), important in proliferating cells [54,55] and primarily known to play a role in the maturation of ribosomal RNAs, ribosome biogenesis, and polysome formation [56,57]. However, ribosomal dysfunction can also trigger apoptosis, autophagy, cell cycle arrest as well as cellular senescence [58,59,60,61,62] and has been related to several hematological disorders, such as Diamond-Blackfan anemia [63,64]. These results prompted us to explore whether PE might be associated with changes in gene expression that adversely affect erythropoiesis and erythroid maturation. Analyzing the proteomics of the in vitro differentiated erythroid cells suggested changes in pathways important in the metabolism, immune system, protein processing, and export as well as phagosomes. Considering that efficient erythroid differentiation and maturation requires a synchronized regulation of iron, amino acid, and glucose metabolism [34], as well as various signaling pathways [65,66], alterations in these pathways could lead to intracellular changes and ineffective erythroid maturation.

To investigate if the results obtained in our in vitro analyses translated into changes in vivo, we compared the UCB erythroid profiles in PE vs. normotensive pregnancies. No significant differences were observed in the erythroid populations from arterial or venous UCB of male or female fetuses in either group. This observation was in agreement with our earlier results and suggested that disruption of erythroid maturation may contribute to a higher erythroblast count in the UCB from PE pregnancies independent of fetal hypoxia-induced EPO-dependent erythropoiesis [18,19,20,21,24]. It is also important to consider that fetal hypoxia in pregnancy is chronic, rather than acute, and hemolysis is not commonly observed in the fetuses born to PE. Thus, the release of immature erythroblasts into the UCB may have explanations other than those suggested for acute hypoxia or hemolytic anemia [67].

**Figure 7:**
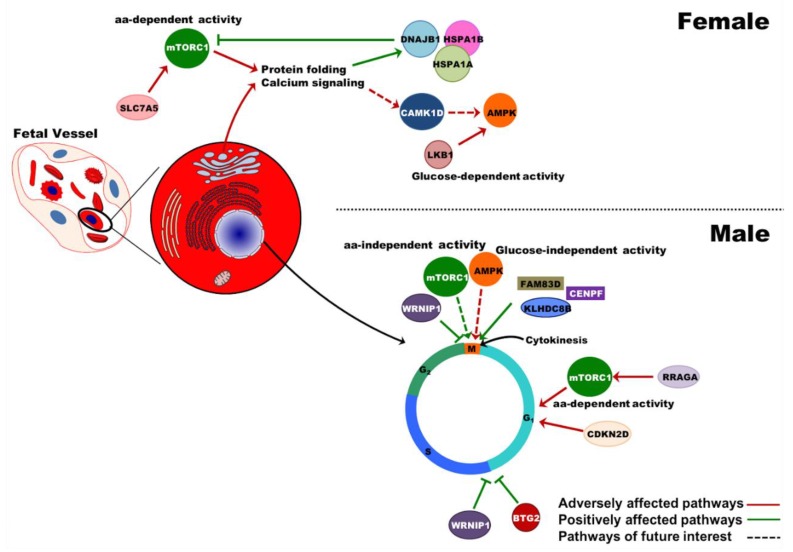
Sex-specific disruption of fetal erythropoiesis in PE. The interplay of the intracellular pathways that could disrupt fetal erythroid maturation has been demonstrated. A magnified erythroid cell with various organelles is used to illustrate the altered pathways in detail. aa: amino acid; SLC7A5 (LAT1): solute carrier family 7 member 5; DNAJB1: DnaJ heat shock protein family (Hsp40) member B1; HSPA1A: heat shock protein family A (Hsp70) member 1A; HSPA1A: heat shock protein family A (Hsp70) member 1B; CAMK1D (CKLiK): calcium/calmodulin dependent protein kinase ID; LKB1 (STK11): serine/threonine kinase 11; AMPK: AMP-activated protein kinase; mTORC1: mammalian target of rapamycin complex1; WRNIP1: Werner helicase interacting protein 1; FAM83D: family with sequence similarity 83 member D; CENPF: centromere protein F; KLHDC8B: kelch domain containing 8B; RRAGA: Ras related GTP binding A; CDKN2D: cyclin dependent kinase inhibitor 2D; BTG2: BTG anti-proliferation factor 2.

To confirm any mechanisms that could affect fetal erythroid maturation and enucleation in PE, the transcriptome of UCB erythroid cells were analyzed. Clustering of the male and female erythroid cells based on transcriptome analysis indicated that the expression of some genes located on both autosomal and sex chromosomes varied between the sexes, specifically upregulating the RNA transcription pathway and several mitochondrial factors in the male fetuses. Comparing the male fetuses among the PE and the normotensive groups indicated a decrease in DNA repair, convergent extension, protein ubiquitination, and vesicle trafficking, as well as deregulation in the cell cycle. For instance, lower CDKN2D (p19) and RRAGA, two regulators of cell cycle G1 progression [68], and amino acid-dependent mTORC1 activity [69,70] were associated with increased BTG2 and WRINP1 that regulate G1/S transition [71] and G1/S or G2/M arrest [72], respectively. Along with these changes, inhibitors of RNA transcription and several genes important in cell cycle S and mitosis phases were upregulated (Supplementary Table S3). Interestingly, KLHDC8B, a factor that safeguards the cell against mitotic errors and nuclear abnormalities, was also increased [73,74]. Due to the absence of significant differences in UCB erythroid populations between PE and normotensive male fetuses, the changes in gene expression could not be explained by elevated late basophilic or polychromatic erythroblasts [75], which express high levels of mitosis-related genes. Thus, the gene expression alterations observed in PE samples from male fetuses may be related to regulation of the cell cycle via mammalian target of rapamycin (mTOR) and AMP-activated protein kinase (AMPK) pathways [76] (Figure 7). Considering their role in regulating cell division, growth, or autophagy [77], altered phosphorylation and imbalance in AMPK/mTOR pathways can lead to a defective cell cycle and also erythroid maturation [78,79,80,81].

Among the female fetuses, decreased metabolism and increased cellular response to stress were the major pathways altered in the PE UCB erythroid cells. Genes that were important in protein processing and calcium homeostasis in Golgi, as well as calcium/calmodulin-dependent protein kinase 1D (CAMK1D) were downregulated in PE. The CAMK1D has been shown to control calcium-induced apoptosis in serum-deprived erythroleukemia cells in vitro [82]. Also, an upstream inducer of AMPK (STK11/LKB1) and amino acid-transporter required for mTORC1 activity (SLC7A5) [83] were lower in UCB erythroid cells from female fetuses born to PE pregnancies. These results along with the upregulated heat shock proteins imply a disturbance in protein processing that might affect cell maturation and survival (Figure 7). Considering that the activity of mTOR and AMPK proteins are regulated by phosphorylation at protein levels, future experiments are required to confirm and evaluate possible phosphorylation changes in erythroblasts in both male and female fetuses from PE pregnancies to indicate any sex-specific associations.

Interestingly, a surface glycoprotein was differentially expressed in each sex group in PE vs. normotensive UCB erythroid cells. Among the male fetuses, the expression of CD99, a glycoprotein associated with the Xg blood group, was upregulated in PE cases. While CD99 is located on the pseudoautosomal areas of sex chromosomes, its level of expression varies during development and based on sex [84]. On the other hand, the female fetuses from PE pregnancies indicated a significant decrease in expressing RP11-342M1.3, which is an antisense to erythroblast membrane associated protein (ERMAP), the surface glycoprotein known for the Scianna blood group [84]. Gene regulation by antisense expression can take place at different layers [85]. Considering that the expression of ERMAP was not significantly different between female PE vs. normotensive erythroid cells, it seems very likely that RP11-342M1.3 has a trans-regulatory effect on ERMAP mRNA. Other candidate genes for future studies include SLC25A6, MTRNR2L1, and CD36 that were determined in various analyses to be differentially expressed in PE and in a sex-specific manner. Further studies are required to confirm the possible sex-specific effects and outcomes of particular changes in fetal erythropoiesis in PE pregnancies.

There is a rising interest in evaluating the effect of various pregnancy complications on fetal development. Long-term analyses of PE pregnancies demonstrate several health problems among the offspring [86], with behavioral and cognitive dysfunction [87] having been a main focus. On the other hand, more studies are shedding light on the significance of fetal sex in regulating various aspects of a pregnancy, from maternal adaptation [41] to placental and fetal gene expression [88,89] and metabolism [90]. Sex-specificity of the placenta function and structure [91] as well as fetal and placental metabolism [90] have been suggested to underlie the higher vulnerability of male fetuses to pregnancy complications, such as obesity or PE [46,92]. The data in this study suggests that besides the nervous system, PE also affects the development of the fetal hematopoietic system. This is mainly through the triggering of transcriptional and proteomic changes in fetal HSPCs and erythroblasts that may disrupt erythroid maturation, explaining the higher UCB erythroblast count in these pregnancies. Our work also suggests that the changes observed in the molecular pathways are more severe among the male compared to the female fetuses. This is in line with higher adverse outcomes in pregnancies with male fetuses [39,40], as well as an increased risk of diseases of the blood and blood-forming organs, such as anemia, among the male children born to PE mothers [93]. 

## 4. Materials and Methods

### 4.1. Ethical Approval and Sample Collection

The study with identification number Dnr 2014/191 was approved on 24 April 2014 by the Lund Regional Ethics Committee Review Board (EPN) for studies on human subjects at Lund University and Skåne University Hospital, Lund, Sweden. Collection of UCB from normotensive and PE pregnancies was performed following both Caesarean and vaginal deliveries at Skåne University Hospital, after written informed consent from patients. All the experiments were performed in accordance with relevant guidelines and regulations. Preeclampsia was defined as blood pressure ≥140/90 mmHg and proteinuria ≥300 mg/L according to ISSHP definition [2]. A summary of the clinical condition of the patients included in this study and the subsequent experiments performed is available in Table 2 (details in Supplementary Table S4). The UCB was collected in flasks containing 10 mL Dulbecco’s Modified Eagle’s Medium, 10% fetal bovine serum (FBS), 100 IU/mL penicillin, 100 μg/mL streptomycin (Gibco^®^, Stockholm, Sweden) and 25 IU/mL heparin (Vianex S.A., Athens, Greece). The samples were stored at 4 °C and processed within 4 h after sampling.

### 4.2. Mononuclear Cell Isolation from the UCB

Total mononuclear cells (MNCs) were isolated from UCB using the density centrifugation media Ficoll-Paque PLUS (GE Healthcare Life Sciences, Uppsala, Sweden) according to the manufacturer’s protocol. In brief, the total UCB was mixed 1:1 (*w/v*) with wash buffer containing 1× phosphate buffered saline (PBS), 2% FBS and 2 mM EDTA. Each sample was carefully laid upon Ficoll-Paque PLUS before centrifugation at 400× *g* for 30 min at room temperature (RT). After the centrifugation, the interphase layer containing the MNCs was retrieved and mixed with ice-cold Iscove′s Modified Dulbecco’s Medium (IMDM), 10% FBS in 1:2 (*w/v*) (Gibco^®^). The MNC suspension was centrifuged and rinsed in wash buffer for erythroid profile analysis or CD34^+^ cell isolation.

### 4.3. Isolation of UCB CD34^+^ Cells

Using the human CD34 MicroBead Kit (Miltenyi Biotec, Lund, Sweden), the UCB CD34^+^ cells were isolated according to the manufacturer’s protocol. In summary, the MNCs were incubated with FcR Blocking Reagent and CD34 MicroBeads at 4 °C for 30 min followed by rinse and centrifugation at 300× *g* at 4 °C for 10 min. The cell suspension was filtered at 40 μm and CD34^+^ cells were magnetically selected on LS columns (Miltenyi Biotec, Lund, Sweden). The CD34^+^ cells were either resuspended in 10% Dimethyl sulfoxide (DMSO) freezing medium, stored at −80 °C for at least 24 h and transferred to liquid nitrogen tank for later in vitro cell culture assays, or were stained and analyzed for surface adhesion molecules using flow cytometry.

### 4.4. Flow Cytometric Analysis of SAM Expression on UCB HSPCs

The UCB HSPCs were detected by flow cytometry as CD34^+^ CD45^+^ cells and the expression of 6 different SAMs was analyzed using phycoerythrin-conjugated mouse anti-human antibodies (Table 1) [49,94,95,96,97]. Six separate suspensions of CD34^+^-enriched cells were prepared and each was incubated for 30 min on ice with appropriate amounts of antibodies (1:25) specific for CD34 (CD34-phycoerythrin/Cy7) and CD45 (CD45-FITC) in combination with one of the SAMs. After rinse in wash buffer, the cells were analyzed using a BD FACSAria™ I. Spectral compensation was carried out using VersaComp Antibody Capture Beads kit (Beckman Coulter, Bromma, Sweden) and the gates were set based on unstained and fluorescent minus one controls. 7AAD at 10 μg/mL (Sigma Aldrich, Stockholm, Sweden) was used as a viability marker. All viable CD34^+^ CD45^+^ cells from each sample were sorted into a tube and used for RNA extraction. Data analysis was performed using FlowJo (V.10.0.8. Ashland, OR, US).

### 4.5. Flow Cytometric Analysis of UCB Stem Cells and Colony Formation Assay 

Frozen CD34^+^ enriched cells were thawed and stored with FcR Blocking Reagent (Miltenyi Biotec) at 4 °C for 30 min. The cells were then stained as described above, with the following antibodies: CD34-phycoerythrin/Cy7, CD38-APC, CD45RA-FITC, and CD90-BV421 (1:25, BD Biosciences, San Jose, CA, US). After performing spectral compensation and setting the gates as mentioned earlier, the UCB HSC population was analyzed by flow cytometry (Table 1) and a total of 15000 viable CD34^+^ cells were collected from each sample using fluorescent activated cell sorting (FACS). The cells were mixed with Cell Resuspension Solution (R&D Systems, Oxon, Sweden) and Human Methylcellulose Complete Media containing EPO, Granulocyte macrophage colony-stimulating factor (GM-CSF), Interlukin-3 (IL-3), and Stem Cell Factor (SCF) (HSC003, R&D Systems). 500 cells/well were plated in triplicate in 6-well plates and placed in a humid chamber incubated at 37 °C with 5% CO2. Burst forming units-erythroid (BFU-Es) were counted in each well after 14 days of culture as indicated by R&D Systems (https://www.rndsystems.com/resources/protocols/human-colony-forming-cell-cfc-assay-using-methylcellulose-based-media).

### 4.6. RNA Extraction and cDNA Subtractive Hybridization

The sorted viable CD34^+^ CD45^+^ cells from aforementioned SAM expression analysis were used for total RNA extraction was performed by lysing the cells in Trizol (Ambion, Naugatuck, CT, US). Following addition of chloroform to the lysate, the aqueous phase was mixed with 70% ethanol and transferred to RNeasy Mini spin columns (Qiagen, Hilden, Germany). Total RNA preparation was completed according to the manufacturer’s protocol. Poly A^+^ RNA purification was performed using Oligotex Direct mRNA mini kit (Qiagen) following the manufacturer’s instructions. Subtractive hybridization was carried out using the Clontech^®^ PCR-Select™ Differential Screening kit (Takara Bio USA, Inc., Mountain View, CA 94043, USA) according to the manufacturer’s protocol. The poly A^+^ RNA from PE and normotensive groups were respectively used as tester and driver for cDNA synthesis. Enriched tester-specific amplicons from the second round of subtraction were ligated into pGEM-T Easy Vector System I (Promega, Madison, WI, US) for insert sequencing (Beckman Coulter Genomics, Bishop’s Stortford, United Kingdom). All retrieved sequences representing genes unique to the tester (PE) population compared to the normotensive population were blasted using BLAST analysis on NCBI (https://blast.ncbi.nlm.nih.gov/Blast.cgi).

### 4.7. Quantitative Proteomic Analysis

Proteomic analysis was performed at the Proteomics Core Facility at Sahlgrenska Academy, University of Gothenburg. For this purpose, the in vitro differentiated erythroid colonies were collected from the colony formation assay. The colonies were rinsed in PBS twice to remove any residues from the culture. The cell pellets were stored at −70 °C prior to lysis and protein extraction. The sample preparation and liquid chromatography-mass spectrometry process were carried out as explained in the Supplementary Materials and Methods [98]. Data analysis was performed using Proteome Discoverer version 1.4 (Thermo Fisher Scientific, Stockholm, Sweden) against the Human Swissprot Database version March 2017 (Swiss Institute of Bioinformatics, Switzerland). Mascot 2.5 (Matrix Science, London, UK) was used as a search engine with precursor mass tolerance of 5 ppm and fragment mass tolerance of 200 mmu. Tryptic peptides were accepted with zero missed cleavage and variable modifications of methionine oxidation, cysteine alkylation and fixed modifications of N-terminal TMT-label and lysine TMT-label were selected. The detected peptide threshold in the software was set to false discovery rate (FDR) ≤0.01 by searching against a reversed database. Identified proteins were grouped by sharing the same sequences to minimize redundancy. Reporter ion intensities were quantified in MS2 spectra at Minimum Quan Value Threshold set to 2000. The resulting ratios were normalized in the Proteome Discoverer 1.4 on the median protein value of 1.0 in each sample. Heat maps were generated using XLSTAT software.

### 4.8. Fluorescent-Activated Sorting of Erythroblasts from the UCB

To block Fc receptors, the isolated MNCs were incubated with FcR Blocking Reagent (Miltenyi Biotec) at 4 °C for 30 min. The cells were rinsed and pelleted 300× *g* at 4 °C. After resuspension and filtering through a 50 μm cup-shaped filter (BD Biosciences), the cells were stained for different surface markers using the following mouse anti-human antibody (ab)-conjugation set: CD45-FITC [1:25], CD235a/Glycophorin A (GPA)-BV421 [1:100] (BD Biosciences), CD49d/Integrin alpha 4- phycoerythrin/Cy7 [1:300] (BioLegend, Täby, Sweden) and CD233/Band 3-phycoerythrin [1:300] (Bristol Institute for Transfusion Sciences). After 30 min incubation on ice, the cells were rinsed and analyzed by a BD FACSAria™ I. 7AAD (Sigma Aldrich, Stockholm, Sweden) at a final concentration of 10 μg/mL was used as a viability marker. Spectral compensation was carried out using VersaComp Antibody Capture Beads kit (Beckman Coulter) and the gates were set based on unstained and fluorescent minus one controls. The data analysis was performed using FlowJo (version 10.0.8).

Considering the few numbers of the early-stage erythroid precursors, all the cells from proerythroblasts (CD45^−^ GPA^+^ CD49d^hi^ Band 3^−^) to reticulocytes (CD45^−^ GPA^+^ CD49d^lo^ Band 3^+^) were pooled together for each arterial and venous UCB sample. A total of 10^6^ cells were sorted and collected for each sample. The cells were centrifuged at 300× *g* for 10 min, lysed in 350 μL of Buffer RLT from AllPrep DNA/RNA/Protein Mini Kit (Qiagen) according to the manufacturer’s protocol and stored at −80 °C until later processing.

### 4.9. RNA Extraction, Library Preparation and Quality Check

All samples were thawed on ice and processed for RNA extraction using AllPrep DNA/RNA/Protein Mini (Qiagen) according to manufacturer’s instructions. All centrifugations were performed at RT at 9000× *g* and the RNA was eluted in 50 μL RNase-free water. RNA samples were frozen at −80 °C and thawed for quality check and library preparation. RNA integrity was analyzed using Agilent RNA 600 Nano Kit (Agilent Technologies, Santa Clara, CA, US) on a Bioanalyzer 2100 (Agilent^TM^, Santa Clara, CA, US) according to the manufacturer’s instructions. All the samples indicated an RNA integrity number (RIN) ≥ 9 and were used for library preparation and amplification by the QuantSeq 3′ mRNA kit (Lexogen^TM^, Vienna, Austria) according to the manufacturer’s protocol. Quality and sequence length of the libraries were assessed by Fragment analyzer using the High sensitivity NGS fragment analysis kit (Advanced Analytical Technologies, Inc., Heidelberg, Germany), while concentration evaluation was performed by BioTek™ Synergy™ 2 (BioTek Instruments, Inc., Winooski, VT, US) microplate reader using Quant-iT^TM^ PicoGreen^TM^ dsDNA Assay Kit (Invitrogen^TM^, Carlsbad, CA, US) for a low-range assay. The RNA library was sequenced in SE50bp mode on an Illumina HiSeq 2500 (Illumina, San Diego, CA, US).

### 4.10. Bioinformatics Analysis

Using random primers, the Lexogen Quantseq kit (Lexogen) generates fragments that end with a poly A tail. Since this random primer may introduce mistakes, the first 11bp of all reads were trimmed. Poly A tails and adapters at the end of each read were trimmed, along with basic quality trimming. Only reads longer than 20 bps were kept for further processing. All trimming and filtering steps were done using bbduk of bbtools 36.84 [99]. Reads were mapped to the Human genome (hg38) using STAR 2.5 [100]. Bam file modifications were done using elprep 2.5 [101]. Htseq 0.6.1p1 [102] was used to count the number of mapped reads per known gene. The gene definitions of Ensembl 87 were used. Reads were only considered in the counting process if the mapping quality was equal to or higher than 10, the strand of the read was the same strand as the gene and the read was not mapped in overlapping gene definitions (the union option). Differential expression analysis was performed using tools DESeq2 [103] and EdgeR [104,105]. The results of these tools were merged. Only genes that were significant with an FDR <0.1 in both tools were considered for further pathway analysis.

### 4.11. Pathway Analysis 

Protein-protein interaction prediction and gene set enrichment analysis (GSEA) were performed by String (V.10.0) [106] and ConsensusPathDB (CPDB) [107] on UCB HSPCs and in vitro differentiated erythroid cells. The link between the genes, human phenotype and diseases were evaluated by performing functional gene list enrichment using ToppFun analysis from ToppGene Suite [108]. Gene ontologies were recovered from Gene, NCBI (https://www.ncbi.nlm.nih.gov/gene/).

### 4.12. Statistical Analysis

To calculate the protein fold change percentage (FC%), the average value of the normalized protein ratio of the PE group was divided over that of the normotensive samples. Also, the normalized protein ratios were used in the Student’s *t*-test to calculate the statistical significance of the observed FC. Considering the low variability and high sensitivity of the TMT-MS, a FC ≥ 20% and *p* value ≤ 0.05 was determined as a significant difference in protein expression between PE and normotensive samples. 

GraphPad Prism (version 7, San Diego, CA, US) was used to perform Mann Whitney U Test and to prepare the graphs.

## 5. Conclusions

In conclusion, our results indicate that the intrinsic migration and differentiation capacity of fetal HSPCs does not alter significantly in PE pregnancies. However, PE is associated with transcriptional and proteomic changes in fetal erythroid cells that may disrupt erythroid maturation, particularly in male fetuses.

## Figures and Tables

**Table 1:** The markers used in flow cytometry analyses.

To Recognize	Markers/Profile
Hematopoietic stem/progenitor cells (HSPCs)	CD34^+^ (clone 581) CD45^+^ (clone HI30)
Surface adhesion molecules (SAMs)	CD44 (clone 515) CD49d (clone 9F10) CD49e (clone IIA1) CD184 (clone 12G5) CD11a (clone HI111) CD62L (polyclonal)
Hematopoietic stem cells (HSCs)	CD34^+^ (clone 581) CD38^lo^ (clone HIT2) CD45RA^−^ (clone HI100) CD90^+^ (clone 5E10)
Erythroid cells (Flow cytometry) from proerythroblasts to mature erythrocytes	CD45^−^ (clone HI30) GPA^+^ (clone HIR2) Band 3 (clone BRIC6) CD49d (clone 9F10)

**Table 2:** Clinical characterization of all the included patients with respect to the experiments.

Analysis	N	Pregnancy Condition	Gestational Age (Weeks)	Comments
SAM expression on UCB HSPCs	10	Normotensive	36–42	These samples were also used for cDNA subtractive hybridization.
	5	PE	36–39	
Colony formation assay	8	Normotensive	38–40	Performed in two sets of individual experiments, with three technical replicates in each set.
	7	PE	30–41	
Quantitative proteomic analysis	5	Normotensive	38–40	The colonies were obtained from the colony formation assay.
	5	PE	37–41	
UCB erythroid profile and transcriptome analysis	7	Normotensive	36–40	
	6	PE	36–40	

## Supplementary Materials

Supplementary materials can be found at https://www.mdpi.com/1422-0067/20/8/2038/s1. Reference [109] is cited in the supplementary materials.

## Abbreviations

PE	Preeclampsia/preeclamptic
UCB	Umbilical cord blood
HS(P)Cs	Hematopoietic stem (progenitor) cells
SAM	Surface adhesion molecule
mTORC1	mammalian target of rapamycin complex 1
AMPK	AMP-activated protein kinase
FDR	False discovery rate
DE	Differentially expressed
