# Supplementary figures and images for: Preeclampsia is Associated with Sex-Specific Transcriptional and Proteomic Changes in Fetal Erythroid Cells
Source: Int J Mol Sci. 2019 Apr 25;20(8):2038. doi: 10.3390/ijms20082038 (PMC6514549; doi:10.3390/ijms20082038)

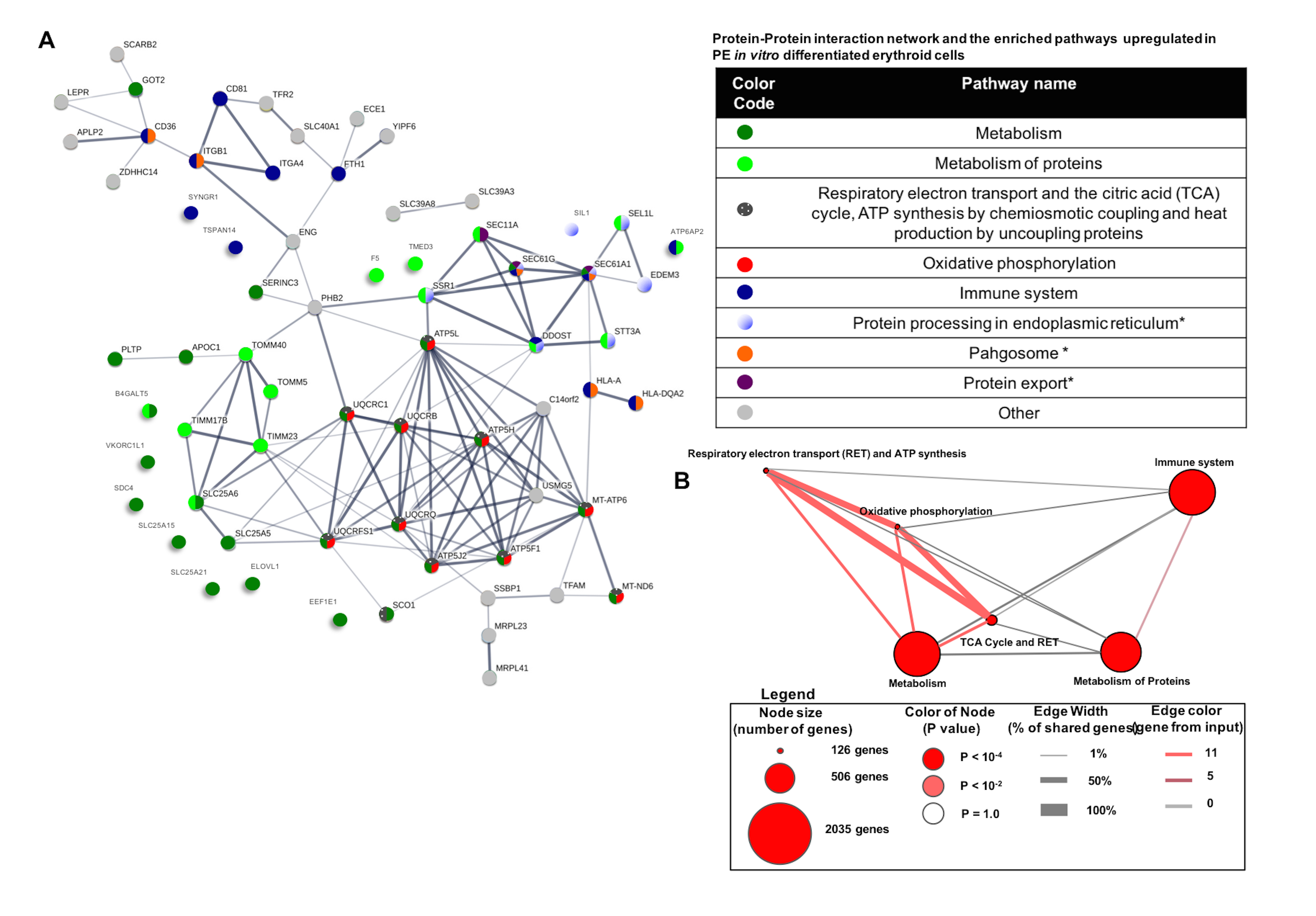

Supplement: Supplementary file 1 [file ijms-20-02038-s001.zip › Supplementary Figure S2- Masoumi.tiff]

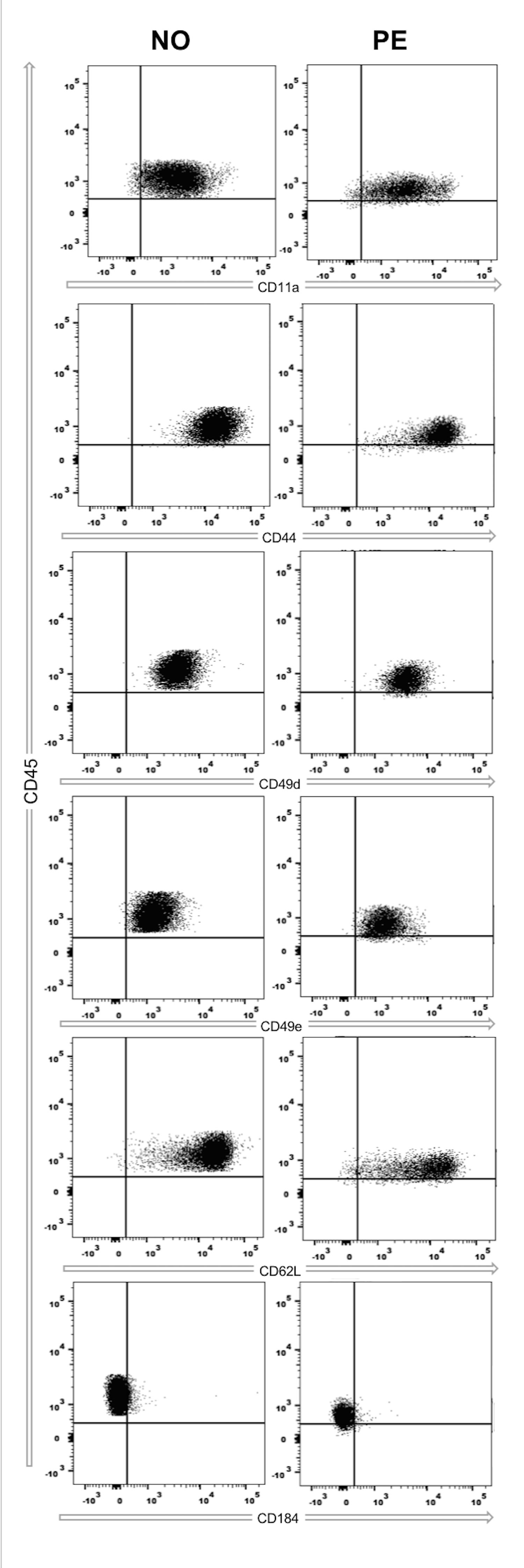

Supplement: Supplementary file 1 [file ijms-20-02038-s001.zip › Supplementary Figure S1- Masoumi.tiff]

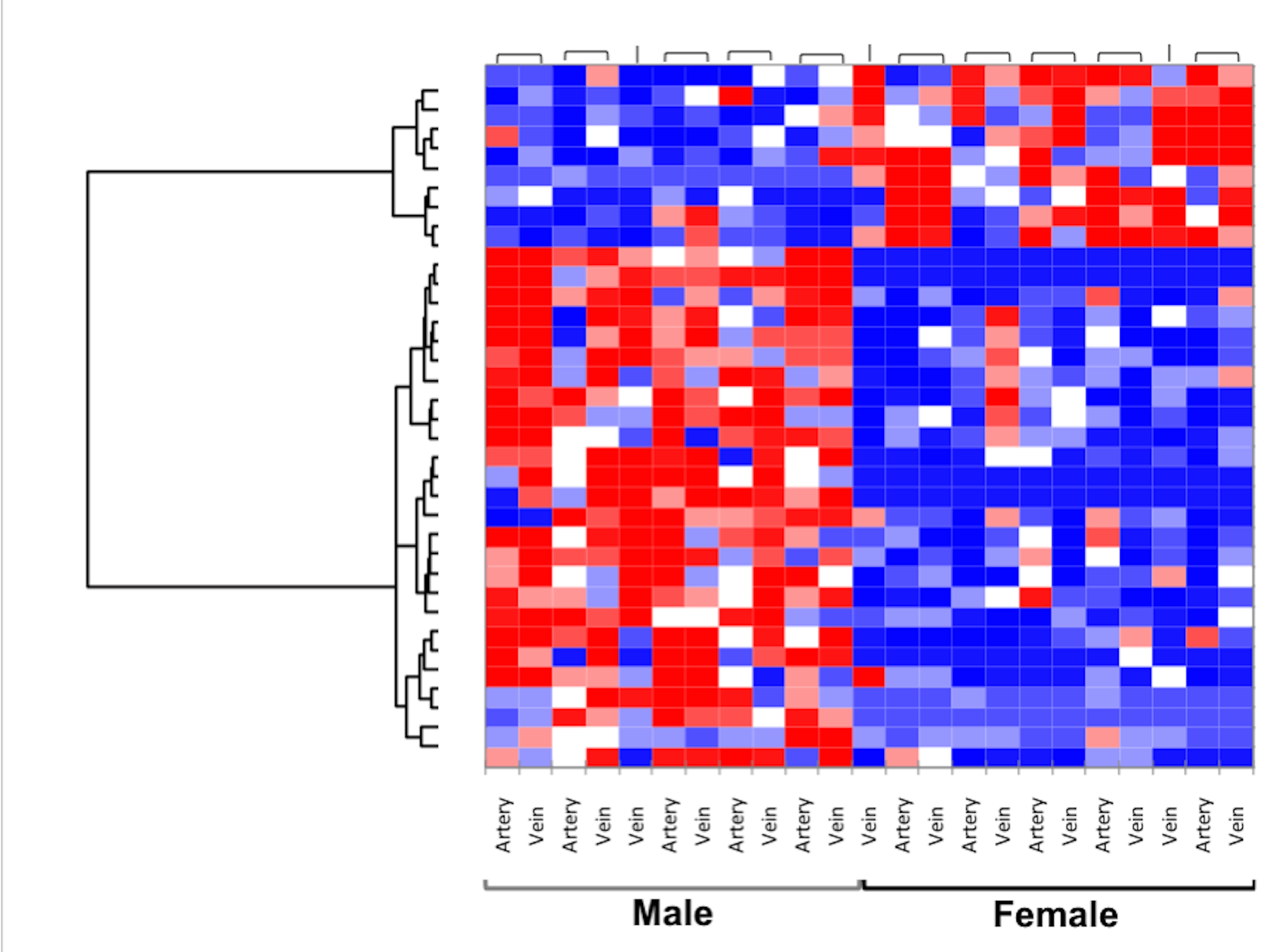

Supplement: Supplementary file 1 [file ijms-20-02038-s001.zip › Supplementary Figure S3- Masoumi.tiff]
