# Supplementary material for: Preeclampsia is Associated with Sex-Specific Transcriptional and Proteomic Changes in Fetal Erythroid Cells
Source: Int J Mol Sci. 2019 Apr 25;20(8):2038. doi: 10.3390/ijms20082038 (PMC6514549; doi:10.3390/ijms20082038)
Supplement: Supplementary file 1 [file ijms-20-02038-s001.zip › Supplementary information.doc]

Preeclampsia is Associated with Sex-Specific Transcriptional and Proteomic Changes in Fetal Erythroid Cells

Zahra Masoumi ^1,^*, Gregory E. Maes ^2,3^, Koen Herten ^2,4^, Álvaro Cortés-Calabuig ^4^,
Abdul Ghani Alattar ^5,6^, Eva Hanson ^1^, Lena Erlandsson ^1^, Eva Mezey ^7^, Mattias Magnusson ^6^,
Joris R Vermeesch ^2,3,4^, Mary Familari ^8,†^ and Stefan R Hansson ^9,†^

^1^	Division of Obstetrics and Gynecology, Department of Clinical Sciences Lund, Lund University, Klinikgatan 28, 22184 Lund, Sweden; [eva.hansson@med.lu.se](mailto:eva.hansson@med.lu.se) (E.H.); [lena.erlandsson@med.lu.se](mailto:lena.erlandsson@med.lu.se) (L.E.);

^2^	Laboratory for Cytogenetics and Genome Research, Center for Human Genetics, KU Leuven, Herestraat 49, 3000 Leuven, Belgium; [gregory.maes@kuleuven.be](mailto:gregory.maes@kuleuven.be) (G.E.M.); [koen.herten@kuleuven.be](mailto:koen.herten@kuleuven.be) (K.H.); [alvaro.cortes@uzleuven.be](mailto:alvaro.cortes@uzleuven.be) (A.C.-C.); [joris.vermeesch@uzleuven.be](mailto:joris.vermeesch@uzleuven.be) (J.R.V.);

^3^	Department of Human Genetics, Centre for Human Genetics, University Hospital Leuven, KU Leuven, Herestraat 49, 3000 Leuven, Belgium

^4^	Genomics Core, UZ Leuven, Herestraat 49, 3000 Leuven, Belgium

^5^	Department of Hematology and Transfusion Medicine, Lund University, Klinikgatan 28, 22184Lund, Sweden; abdul_ghani.alattar@med.lu.se

^6^	Department of Molecular Medicine and Gene Therapy, Lund University, Sölvegatan 17, 22184 Lund, Sweden; [mattias.magnusson@med.lu.se](mailto:mattias.magnusson@med.lu.se);

^7^	Adult Stem Cell Section, National Institute of Dental and Craniofacial Research, National Institutes of Health, 9000 Rockville Pike, Bethesda, MD 20892, USA; MezeyE@nidcr.nih.gov

^8^	School of Biosciences, Bldg 4, University of Melbourne, Parkville 3010, Australia; [m.familari@unimelb.edu.au](mailto:m.familari@unimelb.edu.au);

^9^	Lund University, Skåne University Hospital, Obstetrics and Gynecology, Department of Clinical Sciences Lund, Klinikgatan 28, 22184 Lund, Sweden; [stefan.hansson@med.lu.se](mailto:stefan.hansson@med.lu.se);

*	Correspondence: e-mail: [zahra.masoumi@med.lu.se](mailto:zahra.masoumi@med.lu.se); Tel.: +46 (46) 222 30 27

^†^	These authors contributed equally to this work.

1. **Supplementary Materials and Methods**

*1.1. Protein digestion, peptide labelling and fractionation for quantitative proteomic analysis*

Cell pellets were lysed in 2% SDS, 50 mM Triethylammonium bicarbonate (TEAB) buffer during mixing for 1h. Protein concentration was determined using Pierce™ BCA Protein Assay (Thermo Fisher Scientific, Stockholm, Sweden) and the Benchmark Plus microplate reader (Bio-Rad, Sundyberg, Sweden) with BSA solutions as standards. Aliquots of 20 µg of total protein from each sample and 20 µg from a reference pool containing equal amounts of all samples were digested with trypsin using the filter-aided sample preparation (FASP) method [98]. In summary, protein samples were reduced with 100 mM dithiothreitol at 60°C for 30 min, transferred to 30 kDa MWCO Nanosep centrifugal filters (Pall Life Sciences, NY, US), rinsed with 8M urea solution repeatedly prior to alkylation with 10 mM methyl methanethiosulfonate in digestion buffer (50 mM TEAB and 1% sodium deoxycholate (SDC). Digestion was performed by addition of Pierce mass spectrometry (MS) grade Trypsin protease (0.20 µg, Thermo Fisher Scientific) in the digestion buffer, at a ratio of 1:100 relative to protein amount, and overnight incubation at 37°C. This was followed by another 2-h digestion after additional portion of trypsin was added to the sample. The peptides were collected by centrifugation at 12000 x g and were labelled using TMT 10-plex isobaric mass tagging reagents (Thermo Fisher Scientific) according to the manufacturer’s instructions. The labelled samples were combined into two separate TMT-sets each containing five samples and the reference. Sodium deoxycholate was removed by acidification with 10% TFA.

The two TMT-sets were fractionated into 40 fractions with basic reversed-phase chromatography (bRP-LC) using a Dionex Ultimate 3000 UPLC system (Thermo Fischer Scientific). Peptide separations were performed using a reversed-phase XBridge BEH C18 column (3.5 μm, 3.0x150 mm, Waters Corporation) and a linear gradient from 3% to 40% solvent B over 17 min followed by an increase to 100% solvent B over 5 min. Solvent A was 10 mM ammonium formate buffer at pH 10.00 and solvent B was 90% acetonitrile, 10% 10 mM ammonium formate at pH 10.00. The fractions were combined into 20 fractions, dried and reconstituted in MilliQ water containing 3% acetonitrile, 0.2% formic acid for nano-liquid chromatography- mass spectrometry (NanoLC-MS) analysis.

1.2. *NanoLC MS analysis and database search*

The fractions were analysed on a QExactive HF mass spectrometer interfaced with Easy-nLC1200 liquid chromatography system (Thermo Fisher Scientific). Peptides were trapped on an Acclaim Pepmap 100 C18 trap column (100 μm x 2 cm, particle size 5 μm, Thermo Fischer Scientific) and separated on an in-house packed analytical column (75 μm x 300 mm, particle size 3 μm, Reprosil-Pur C18, Dr. Maisch) using a linear gradient from 9% to 35% solvent B (diluted in solvent A) over 75 min followed by an increase to 100% solvent B for 5 min at a flow of 300 nL/min. Solvent A was 0.2% formic acid and solvent B was 80% acetonitrile, 0.2% formic acid, both in MilliQ water. The instrument operated in data-dependent mode where the precursor ion mass spectra were acquired at a resolution of 60000, the 10 most intense ions were isolated in a 0.8 Da isolation window and fragmented using collision energy HCD settings at either 28 or 50. MS2 spectra were recorded at a resolution of 60000, charge states 2 to 4 were selected for fragmentation and dynamic exclusion was set to 20 s with 10 ppm tolerance. The results were used for data analysis using Proteome Discoverer version 1.4 (Thermo Fisher Scientific).

2. **Supplementary Figures**

**Supplementary Figure 1.** Flow cytograms for the analysis of expression of adhesion molecules on surface of hematopoietic stem/progenitor cells (HSPCs). The HSPCS (CD34^+^ CD45^+^) were gated as indicated in Fig 1A. These flow cytograms demonstrate the expression of the investigated surface adhesion molecules in the umbilical cord blood HSPCs in both preeclamptic (PE) and normotensive (NO) groups.

**Supplementary Figure 2.** Protein- protein interaction network and the pathway interactions. The protein-protein network for the significantly higher protein in PE vs normotensive samples are indicted (A). The protein interactions were analyzed by String showing medium () to high confidence () interactions. The proteins are labelled according to their presence in each pathway and the disconnected nodes were also added accordingly. The enriched pathways were analyzed by String and CPDB (using KEGG and Reactome pathway databases) at FDR<0.01. The connection between various pathways is also demonstrated (B).

**Supplementary Figure 3.** Gene expression analysis in the arterial and venous UCB erythroid progenitors. The heat map demonstrates the gene expression pattern were similar between arterial and venous UCB samples originating from the same fetus. Artery and vein blood sample from the same origin are indicated by horizontal brackets on the top.

**Supplementary Table 1: The enriched pathway sets in UCB HSPCs in PE.**

| **Pathway name** | **Candidates** | **p-value** | **FDR** | **Pathway source** |
| --- | --- | --- | --- | --- |
| Ribosome - Homo sapiens (human)* | 18 | 1.38e-30 | 2.91e-30 | KEGG |
| Peptide chain elongation | 19 | 1.16e-36 | 4.41e-35 | Reactome |
| Eukaryotic Translation Elongation | 19 | 3.14e-36 | 5.97e-35 | Reactome |
| Selenocysteine synthesis | 18 | 8.04e-34 | 7.37e-33 | Reactome |
| Eukaryotic Translation Termination | 18 | 9.7e-34 | 7.37e-33 | Reactome |
| Nonsense Mediated Decay (NMD) independent of the Exon Junction Complex (EJC) | 18 | 1.68e-33 | 1.07e-32 | Reactome |
| Formation of a pool of free 40S subunits | 18 | 5.72e-33 | 3.11e-32 | Reactome |
| Nonsense Mediated Decay (NMD) enhanced by the Exon Junction Complex (EJC) | 18 | 1.53e-32 | 6.48e-32 | Reactome |
| Nonsense-Mediated Decay (NMD) | 18 | 1.53e-32 | 6.48e-32 | Reactome |
| SRP-dependent cotranslational protein targeting to membrane | 18 | 2.88e-32 | 1.09e-31 | Reactome |
| GTP hydrolysis and joining of the 60S ribosomal subunit | 18 | 3.9e-32 | 1.14e-31 | Reactome |
| L13a-mediated translational silencing of Ceruloplasmin expression | 18 | 3.9e-32 | 1.14e-31 | Reactome |
| 3, -UTR-mediated translational regulation | 18 | 3.9e-32 | 1.14e-31 | Reactome |
| Translation | 19 | 6.97e-32 | 1.8e-31 | Reactome |
| Selenoamino acid metabolism | 18 | 7.09e-32 | 1.8e-31 | Reactome |
| Cap-dependent Translation Initiation | 18 | 1.26e-31 | 2.82e-31 | Reactome |
| Eukaryotic Translation Initiation | 18 | 1.26e-31 | 2.82e-31 | Reactome |
| Metabolism of amino acids and derivatives | 18 | 1.73e-24 | 3.46e-24 | Reactome |
| Metabolism of proteins | 20 | 1.82e-15 | 3.46e-15 | Reactome |
| Metabolism | 20 | 5.95e-13 | 1.08e-12 | Reactome |
| Gene Expression | 19 | 8.73e-13 | 1.51e-12 | Reactome |
| Formation of the ternary complex, and subsequently, the 43S complex | 7 | 1.08e-11 | 1.78e-11 | Reactome |
| Ribosomal scanning and start codon recognition | 7 | 2.72e-11 | 4.31e-11 | Reactome |
| Translation initiation complex formation | 7 | 3.03e-11 | 4.61e-11 | Reactome |
| Activation of the mRNA upon binding of the cap-binding complex and eIFs, and subsequent binding to 43S | 7 | 3.37e-11 | 4.93e-11 | Reactome |
| rRNA modification in the nucleus and cytosol | 2 | 0.00571 | 0.00774 | Reactome |
| rRNA processing in the nucleus and cytosol | 2 | 0.00571 | 0.00774 | Reactome |
| rRNA processing | 2 | 0.00687 | 0.009 | Reactome |

*This pathway was significant in both String and CPDB.

The analysis in CPDB was performed using over-representation analysis in the gene set analysis in human network based on differentially expressed genes obtained by cDNA subtractive hybridization.

**Supplementary Table 2:** The list of all the detected and differentially expressed (DE) proteins from in vitro differentiated erythroid cells determined by TMT-mass spectrometry.

**Supplementary Table 3:** The list of the differentially expressed (DE) genes in UCB erythroid cells from male and/or female fetuses in PE vs normotensive pregnancies.

**Supplementary Table 4: Detailed description of the clinical characteristics of all the included patients respective to the experiments.**

| **Pat#** | **Fetal sex** | **Fetal weight (g)** | **Gestational age**  **(W+D)** | **Percentile ranks**  **(Z score)** | **Growth Classification^*†^** | **Mode of delivery** | **Maternal age** | **Maternal BMI** | **Parity** | **Gravity** | **Pregnancy condition** |
| --- | --- | --- | --- | --- | --- | --- | --- | --- | --- | --- | --- |
|  | | | | | | | | | | | |
| Samples used in SAM expression analysis on UCB HSPCs and cDNA subtractive hybridization: | | | | | | | | | | | |
| P871 | Male | 3490 | 39+4 | 45 (-0.13) | AGA | Vaginal | 35 | 26 | 2 | 2 | Normotensive |
| P875 | Male | 4285 | 40+3 | 88 (1.22) | AGA | Cesarean section | 35 | 21 | 1 | 1 | Normotensive |
| P877 | Female | 4720 | 40+1 | 99.6 (2.65) | LGA | Vaginal | 34 | 22 | 2 | 2 | Normotensive |
| P879 | Female | 4600 | 42+1 | 94 (1.53) | LGA | Vaginal | 35 | 22 | 2 | 2 | Normotensive |
| P880 | Female | 3905 | 41+0 | 62 (0.47) | AGA | Vaginal | 27 | 22 | 2 | 3 | Normotensive |
| P882 | Male | 3755 | 41+3 | 37 (-0.31) | AGA | Vaginal | 24 | 24 | 1 | 1 | Normotensive |
| P884 | Male | 2345 | 36+3 | 94 (1.54) | LGA | Vaginal | 30 | 25 | 2 | 2 | Normotensive |
| P885 | Female | 3495 | 38+2 | 75 (0.71) | AGA | Vaginal | 20 | 19 | 2 | 2 | Normotensive |
| P886 | Male | 4420 | 39+1 | 99 (2.21) | LGA | Vaginal | 24 | 24 | 1 | 1 | Normotensive |
| P887 | Female | 4000 | 42+1 | 58 (0.29) | AGA | Vaginal | 32 | 33 | 1 | 1 | Normotensive |
| P876 | Male | 3790 | 39+2 | 74 (0.68) | AGA | Vaginal | 31 | 22 | 2 | 3 | PE |
| P891 | Male | 3415 | 39+5 | 36 (-0.36) | AGA | Vaginal | 29 | 29 | 2 | 3 | PE |
| P892 | Female | 1925 | 37+3 | 1 (-2.94) | SGA | Cesarean section | 38 | 24 | 1 | 1 | PE |
| P894 | Male | 3805 | 36+1 | 99.7 (2.7) | LGA | Vaginal | 27 | 22 | 1 | 2 | PE |
| P907 | Female | 4100 | 39+2 | 95 (1.66) | LGA | Vaginal | 30 | 34 | 0 | 1 | PE |

| Samples used in colony formation assay (all) and quantitative proteomics analysis (in red): | | | | | | | | | | | |
| --- | --- | --- | --- | --- | --- | --- | --- | --- | --- | --- | --- |
| P909 | Male | 4000 | 40+2 | 73 (0.67) | AGA | Vaginal | 29 | 22 | 0 | 1 | Normotensive |
| P911 | Female | 3160 | 39+6 | 22 (-0.77) | AGA | Vaginal | 29 | 23 | 0 | 0 | Normotensive |
| P916 | Male | 3625 | 40+5 | 38 (-0.31) | AGA | Vaginal | 27 | 42 | 2 | 1 | Normotensive |
| P922 | Male | 3220 | 38+6 | 33 (-0.44) | AGA | Vaginal | 30 | 17 | 0 | 2 | Normotensive |
| P924 | Female | 3240 | 39+6 | 27 (-0.59) | AGA | Vaginal | 36 | 23 | 5 | 9 | Normotensive |
| P925 | Male | 3205 | 39+2 | 25 (-0.66) | AGA | Vaginal | 28 | 20 | 0 | 2 | Normotensive |
| P927 | Female | 3485 | 40+4 | 38 (-0.3) | AGA | Vaginal | 29 | 19.9 | 0 | 2 | Normotensive |
| P928 | Female | 3935 | 39+1 | 91 (1.34) | LGA | Vaginal | 35 | 22.6 | 0 | 1 | Normotensive |
| P899 | Male | 3155 | 38+5 | 30 (-0.53) | AGA | Vaginal | 26 | 30 | 0 | 1 | PE |
| P905 | Female | 1450 | 30+1 | 21 (-0.8) | AGA | Cesarean section | 29 | 19.9 | 0 | 1 | PE |
| P919 | Male | 3380 | 39+0 | 25 (-0.65) | AGA | Vaginal | 33 | 20 | 0 | 0 | PE |
| P923 | Female | 1085 | 30+1 | 1 (-2.61) | SGA | Cesarean section | 21 | 33 | 0 | 1 | PE |
| P926 | Female | 4170 | 41+1 | 84 (0.99) | AGA | Cesarean section | 24 | 38 | 0 | 3 | PE |
| P929 | Male | 3178 | 36+4 | 73 (0.66) | AGA | Cesarean section | 26 | 21 | 0 | 2 | PE |
| P930 | Male | 2574 | 37+0 | 12 (-1.19) | AGA | Vaginal | 34 | 20 | 1 | 3 | PE |

| Samples used in UCB erythroid profile and RNA sequencing analysis: | | | | | | | | | | | |
| --- | --- | --- | --- | --- | --- | --- | --- | --- | --- | --- | --- |
| P946 | Female | 3160 | 37+5 | 54 (0.15) | AGA | Vaginal | 24 | 22 | 0 | 1 | Normotensive |
| P947 | Female | 3880 | 40+0 | 78 (0.81) | AGA | vaginal | 22 | 23 | 0 | 1 | Normotensive |
| P948 | Male | 3235 | 39+6 | 21 (-0.82) | AGA | Vaginal | 27 | 22 | 2 | 3 | Normotensive |
| P952 | Male | 2760 | 39+0 | 6 (-1.57) | SGA | Vaginal | 30 | 24 | 0 | 1 | Normotensive |
| P956 | Male | 3600 | 39+5 | 52 (0.05) | AGA | Vaginal | 29 | 20 | 1 | 2 | Normotensive |
| P957 | Female | 3495 | 39+4 | 53 (0.11) | AGA | Vaginal | 36 | 31 | 1 | 4 | Normotensive |
| P958 | Female | 3305 | 36+5 | 87 (1.11) | AGA | Vaginal | 39 | 22 | 1 | 2 | Normotensive |
| P951 | Male | 2950 | 36+0 | 60 (0.38) | AGA | Vaginal | 36 | 24 | 1 | 2 | PE |
| P954 | Male | 3820 | 39+2 | 75 (0.75) | AGA | Vaginal | 29 | 25 | 1 | 3 | PE |
| P955 | Male | 4390 | 39+4 | 97 (1.89) | LGA | Vaginal | 25 | 26 | 0 | 1 | PE |
| P959 | Female | 3215 | 39+3 | 32 (-0.48) | AGA | Vaginal | 39 | 25 | 0 | 2 | PE |
| P962 | Female | 3125 | 37+5 | 52 (0.06) | AGA | Vaginal | 29 | 19 | 0 | 1 | PE |
| P963 | Female | 3900 | 40+0 | 80 (0.86) | AGA | Vaginal | 35 | 22 | 0 | 1 | PE |

*Growth Classification was based on the Z score calculated as (109). Z score < -1.27 are small for gestational age (SGA), -1.27 < Z score < 1.27 are considered appropriate for gestational age (AGA), and Z score > 1.27 are large for gestational age (LGA).

†The LGA and SGA samples were analyzed for Doppler abnormalities. No abnormalities were observed except in case of P923 where uterine arterial score, blood flow class and pulsality index were 4, 1 and 1.54, respectively, suggesting fetal growth restriction.
